# Osteoid osteoma of the talus: a clinical case report and literature review

**DOI:** 10.1016/j.ijscr.2025.112053

**Published:** 2025-10-13

**Authors:** M. Diop, M. Sow, B. Dembele, M. Daffé, B. Diop, A.D. Sané

**Affiliations:** a Department of Orthopedics and Traumatology, Dalal Jamm Hospital of Guediawaye, Dakar, Senegal; b Department of Orthopedics and Traumatology Regional Hospital of Saint Louis, Senegal

**Keywords:** Case report, Osteoid osteoma, Curettage, Cement, Talus, Bone tumor

## Abstract

**Introduction and importance:**

Osteoid osteoma of the talus is a relatively rare benign tumor. It accounts for 10 to 11 % of all benign bone tumors. It generally affects long bones. In our patient, the initial clinical and radiological presentation led to the suspicion of a giant cell tumor.

**Case presentation:**

The authors report the case of a 29-year-old patient who presented with chronic pain in the foot and ankle. X-ray and CT scan revealed well-defined lytic lesions of the talus. The treatment was surgical. A hollowing followed by the placement of cement was carried out. The diagnosis of osteoid osteoma was confirmed by pathological examination of the curettage product. The evolution was favorable with no sign of recurrence.

**Clinical discussion:**

Treatment for this lesion is primarily surgical. The lesion was managed by curettage followed by cement augmentation. Arthroscopic resection is an increasingly used therapeutic option.

**Conclusion:**

The outcome is usually favorable with a low risk of recurrence.

## Highlights

• Osteoid osteoma of the talus is a relatively rare benign tumor.
• Osteoid osteoma may present atypically and mimic aggressive tumors such as giant cell tumor.
• In our case, the lesion was successfully managed by curettage and cement augmentation.

## 1 Introduction

Osteoid osteoma is a benign osteoblastic tumor, first described by Jaffe [1]. It accounts for 10 to 11 % of all benign bone tumors [2]. It is typically located in the long bones (proximal femur, tibia). Involvement of the bones of the foot and ankle is less common, with the talus accounting for 5 % to 8 % of cases [3]. This tumor often affects young individuals.

The diagnosis is often missed and requires cross-sectional imaging. In this case medical imaging suggested a giant cell tumor but confirmation is always histological. Treatment is essentially surgical.

We report the case of a 29-year-old patient who presented with an osteoid osteoma of the left talus. Curettage followed by filling with cement was performed. Histopathological examination confirmed the diagnosis. At follow-up, the patient reported no pain.

The work has been reported in line with the SCARE criteria [4].

## 2 Observation

A 29-year-old man, student with no reported past medical history. He presented with chronic pain in his left ankle. The pain was intermittent, relieved by common analgesics, and aggravated by walking. The pain is relieved at night.

On clinical examination, the patient walked with a slight limp. There was no ankle swelling. The overlying skin was healthy and ankle mobility was normal. The rest of the examination was unremarkable.

**Fig. 1:**
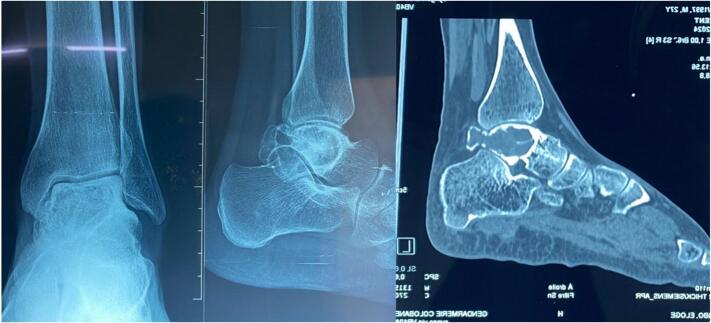
Ankle imaging: A (Ankle radiograph); B (Ankle computed tomography scan).

A standard radiograph of the ankle showed osteolytic and osteosclerotic lesions involving the body of the talus. A computed tomography (CT) scan of the ankle confirmed the presence of lytic lesions with septations (Fig. 1). These lesions were suggestive of a giant cell tumor.

**Fig. 2:**
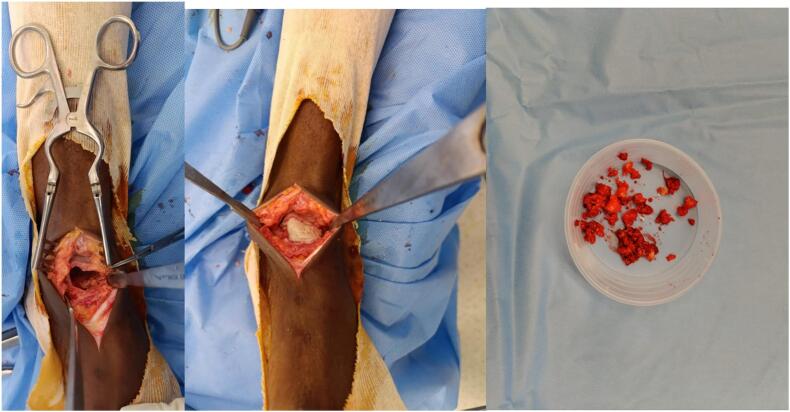
Intraoperative images.

**Fig. 3:**
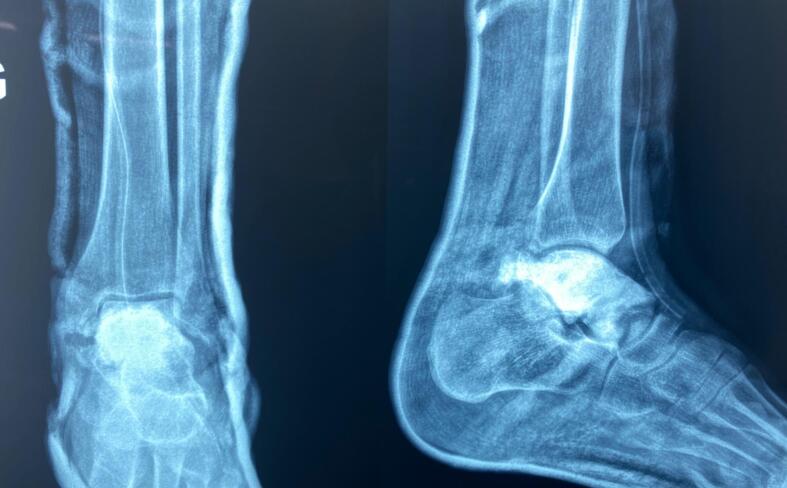
Postoperative radiograph showing the presence of cement.

The patient was placed in the supine position under spinal anesthesia. An anteromedial approach to the ankle was performed. The corticotomy was performed at the junction of the talar neck and body, sparing the articular cartilage. Complete curettage of the tumor was carried out, followed by acrylic cement packing (Fig. 2, Fig. 3). A posterior boot splint was applied.

**Fig. 4:**
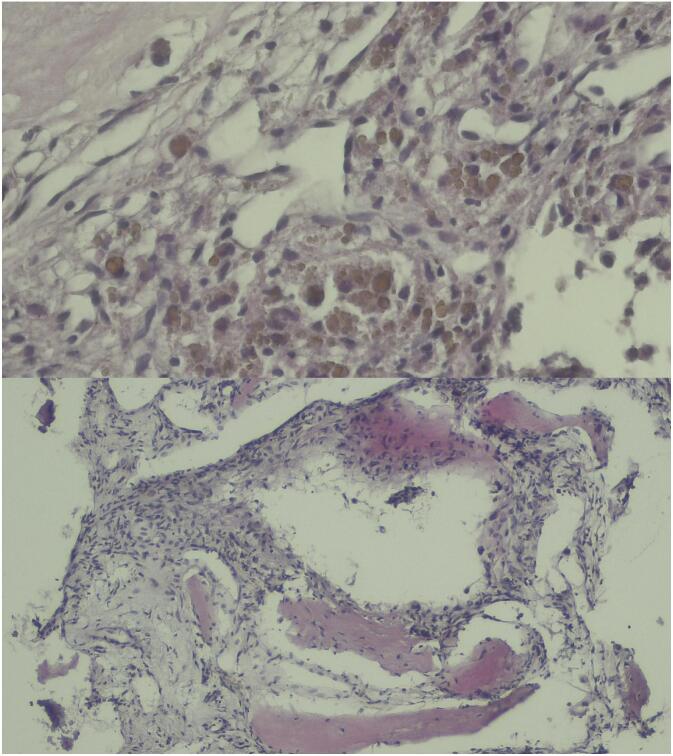
Histopathological images of the curettage specimen.

A histopathological examination of the curettage specimen was requested. The diagnosis of osteoid osteoma was confirmed by histology (Fig. 4).

The patient was followed up regularly. At the 6-month follow-up, the patient reported no pain. There were no signs of local recurrence. The patient had resumed his activities of daily living.

## 3 Discussion

Osteoid osteoma is a benign bone tumor that preferentially affects the long bones [3,5]. It occurs mainly in young individuals (12 years old), with a male predominance [6,7]. Involvement of the foot bones is unusual, occurring in about 8 % of cases. The diagnosis can often be missed. In our context, the diagnostic delay is due to reliance on traditional healers and delayed medical consultation. Pain is the main symptom; it is aggravated by walking and relieved by common analgesics. The classical symptom of nocturnal pain and relief with NSAIDs is reported to occur only in half the cases [8].

Snow et al. [9] showed that there is frequently a diagnostic delay in talar locations.

Standard radiography reveals findings suggestive of osteoid osteoma. This appears as an intracortical lucency surrounded by significant osteosclerosis. The nidus may contain calcifications, reflecting the ossification of the osteoid matrix in long-standing lesions [10]. Thin-slice computed tomography is very reliable for detecting these tumorous lesions. Many authors consider CT the best tool for diagnosing osteoid osteoma of the talus. However, the definitive diagnosis remains histopathological.

Treatment for this lesion is primarily surgical. There is no consensus on the specific type of treatment. According to Muller and Carlioz [11], treatment must meet two requirements: achieve complete excision of the lesion to prevent recurrence, and avoid overly wide resection, which risks weakening the bone segment.

Several techniques have been developed. Typically, open excision is often recommended. It allows for the removal of the entire tumor. We performed an open approach. This approach allowed us to expose the talus, perform debridement and complete curettage of the tumor, and then pack the defect with cement. This option was used because we initially suspected a giant cell tumor. For us, the open treatment was straightforward.

Arthroscopic resection has been proposed by some authors [12,13]. Arthroscopic excision has been found to be a less invasive alterative to open excision for management of osteoid osteoma in the talus [14].

CT-guided percutaneous drilling is of great benefit in the treatment of deep-seated osteoid osteomas. The only major drawback of radiofrequency remains the lack of tissue for histological confirmation of diagnosis.

Osteoid osteoma is a benign tumor that generally has a favorable course. Recurrence is uncommon if the treatment is performed correctly. The outcome for our patient was favorable, with resolution of pain and a return to daily activities.

## 4 Conclusion

Osteoid osteoma located in the foot is uncommon. Osteoid osteoma of the talus can often be missed. Its treatment is primarily surgical and involves several techniques. The definitive diagnosis is always histological. The outcome is usually favorable with a low risk of recurrence.

## Consent

Consent was obtained from the patient.

## Ethical approval

Ethics approval is not required for this manuscript in our institution.

## Funding

No funding for the preparation of this document.

## Author contribution

M.D.: designed the study.

MS, BD, MD, and BD read and corrected the manuscript.

ADS supervised the work.

All authors approved the final version of the manuscript.

## Guarantor

Malick Diop.

## Research registration number

Research registry 10936.

## Declaration of Generative AI and AI-assisted technologies in the writing process

**AI** was not used by author.

## Conflict of interest statement

No conflicts interest in related to this study.
